# Early Warning Method for Regional Water Resources Carrying Capacity Based on the Logical Curve and Aggregate Warning Index

**DOI:** 10.3390/ijerph17072206

**Published:** 2020-03-25

**Authors:** Menglu Chen, Juliang Jin, Shaowei Ning, Yuliang Zhou, Parmeshwar Udmale

**Affiliations:** 1 School of Civil Engineering, Hefei University of Technology, Hefei 230009, China; chenmenglu901127@sina.com (M.C.); jinjl66@126.com (J.J.); ZYL54600@163.com (Y.Z.); 2 Department of Civil and Earth Resources Engineering, Kyoto University, Kyoto 615-8540, Japan; 3 Department of Development and Sustainability, Asian Institute of Technology, Pathumthani 12120, Thailand; udmale@ait.asia

**Keywords:** regional water resources carrying capacity, early warning, logical curve, KLR model, AGA-AHP, Anhui Province

## Abstract

The sustainable utilization of water resources is a significant factor in the development of the national economy and society. Regional water resources carrying capacity (RWRCC) is an appropriate method for evaluating the balance in such utilization. In this paper, we combined time difference correlation analysis and set pair analysis firstly to identify the early warning sign index (EWSI) for RWRCC, and warning limits were determined using a logical curve. Analytic hierarchy process based on the accelerating genetic algorithm (AGA-AHP) method was used to improve the KLR model by determining weights objectively. We took advantage of the new improved model to build the aggregate warning index (AWI). Then, according to the corresponding relationship between EWSI and AWI, the early warning system for regional water resources carrying capacity (EWS-RWRCC) was established, and a case study was carried out in Anhui Province. The results showed there are eight effective EWSI obtained through the early warning analysis process of RWRCC in Anhui Province, among which the repetitive use rate of industrial water and average daily coefficient have a greater impact on AWI. Basically, the EWS-RWRCC can describe RWRCC changes in Anhui Province. From 2006 to 2014, more than half the signal lights in Anhui Province were yellow and orange, which indicated a poor state. It has been proved that the constraints of population, GDP growth and water supply capacity on the utilization of water resources in the future will be further tightened, which should be considered for future monitoring and early warning. The early warning method we used here can be widely applied into other fields; the results will enhance monitoring capacity and scientifically guide regional water resources management.

## 1. Introduction

The global environment has changed rapidly in recent years, in response to the increased demand on natural resources [1]. Because water resources are one of the most important natural resources, their shortage has become a development constraint impeding the economic growth of many countries [2,3]. The importance of capacity building in water management has been widely recognized [4]. Economic development, climate change, and other drivers alter water availability [5], resulting in the increased risk of water security issues. In particular, industrial development without adequate water treatment or recycling will lead to pollution that endangers ecosystems and human health [6]. In light of the complexity and seriousness of water security, scholars all over the world have proposed some solutions [7,8]. Of these, regional water resources carrying capacity (RWRCC) can analyze the potential relationship between water resources and socioeconomic systems, which has become an important research topic in recent years [9,10].

An early warning system (EWS) allows individuals or organizations exposed to hazard so take timely action to avoid or reduce risk and prepare for effective response. Dynamical warning systems can have tipping points at which a sudden shift may occur [11]. The first high-throughput development was the forecasting methodology that seeks to synthesize the factors in economics [12]. With the continuous development of system theory, EWS has gradually infiltrated into safe management of natural resources [13,14]. Meanwhile, its application in water resources management is still in the preliminary stages. Case studies of EWS for water resources have not only been focused on natural disasters like floods and torrential rain but have also been related to water quality and the sustainable utilization of water resources. Today, most EWSs of floods are based either on watershed hydrological models [15] or stochastic rainfall runoff forecast models [16]. These show different features along small or large rivers. Some studies have used satellite remote sensing technologies for early flood warning, which can track the process of flood formation in a large plains region [17]. In a stream, the time interval between rainfall and peak flow may be short, so weather forecasts are required to provide warning in a timely manner. Although some river discharge is sufficient to handle water demand for many years, significant water pollution events still cause frequent disruptions to water supplies. Therefore, monitoring and detecting warning signs of changes in drinking water quality variables play an essential role in ensuring high-quality drinking water and avoiding health-related problems [18]. Many methods, such as data mining [19], instantaneous point source two-dimensional water quality modeling [20], the driving force–pressure–state–influence–response (DPSIR) model [21], the entropy-weight method [22], and the system dynamics (SD) model [23], have been used to predict temporal and spatial variability and trends and to model average pollutant concentration levels and peak values in primary urban drinking water sources.

## 2. Theoretical Framework

**Figure 1:**
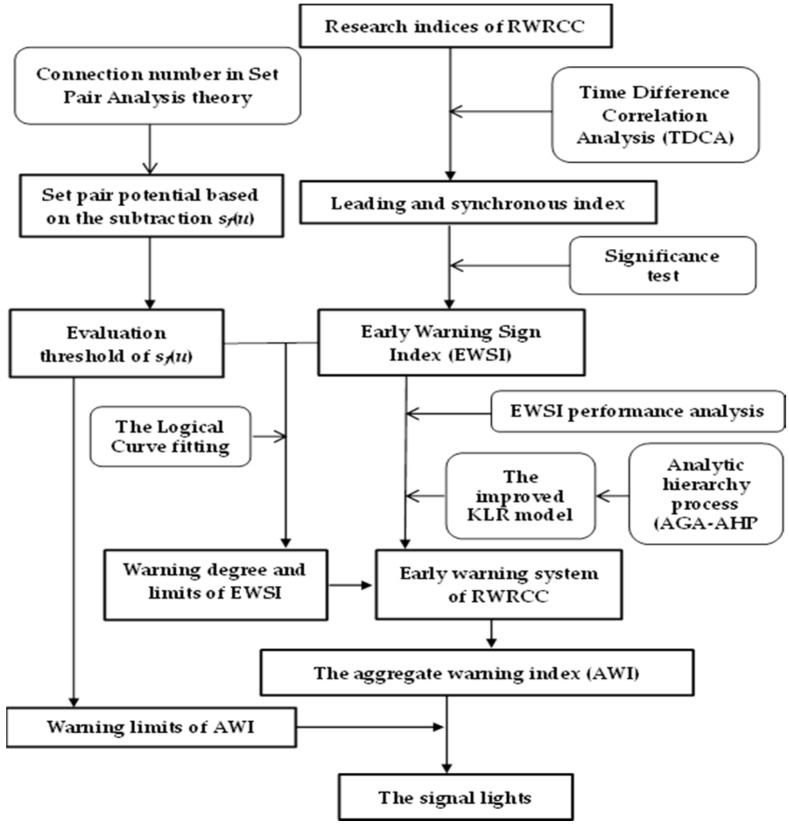
Process of early warning method of regional water resources carrying capacity.

The process of early warning method included the following steps and analytical approaches (Figure 1):

The early warning system for regional water resources carrying capacity (EWS-RWRCC) has become a main tool for sustainability strategy because water resources are an important component of resource-based carrying capacity [24]. Due to the complexity and uncertainty of water resources, current research is far from meeting the needs of regional sustainable development. There are still many shortcomings, such as the lack of a theoretical basis for defining critical warning values and quantitative models for EWS-RWRCC. In this paper, an early warning sign index (EWSI) was identified and index standards were scientifically defined. According to the relationship between EWSI and the aggregate warning index (AWI), a EWS-RWRCC in Anhui Province, China, was established to evaluate the state of water resources.

## 3. Methodology

### 3.1. Identification of EWS for EWS-RWRCC

Time difference correlation analysis (TDCA) is a statistical method for studying the correlations between random variables. It holds that a correlation coefficient between two time series will reach a maximum at a certain point in time [25,26]. This method selects a benchmark index and then moves the research indices forward or backward in time, while calculating the corresponding correlation coefficients. The above process is repeated and a moving period corresponding to the maximum coefficient is the time difference correlation.

According to set pair analysis (SPA) theory, set pair potential based on the subtraction of connection numbers [27,28] can describe the relative deterministic state of objects at the current macro level. We used it as the benchmark index for EWS-RWRCC.

Let the set pair potential based on subtraction be {*s_f_*(*u_t_*) | *t* = 1, 2, …, *n_t_*} [27] in Equation (1):

(1) $$s_{f}\left(u_{t}\right)=\left(a-c\right)\left(1+b\right)$$

(2) $$u_{t}=a+bI+cJ$$

where *n_t_* represents the number of years, *u_t_* represents the connection numbers obtained by RWRCC evaluation. *a*, *b*, *c* ∈ [0, 1]; *a* + *b* + *c* = 1; *a*, *b* and *c* represent degrees of identity, difference, and opposition [10]. *I* is the coefficient of discrepancy and *J* is the opposite coefficient that is generally equal to −1.

Let research indices be {*x*(*i*, *t*) |*I* = 1, 2, …, *n_i_*, *t* = 1, 2, …, *n_t_*}, *n_i_* represents the number of indices, *n_t_* represents the number of years. Therefore, the correlation coefficients between the research index *i* and the benchmark index under moving period *l* are [29]:

(3) $$R\left(i,l\right)=\frac{\sum_{t=t^{\prime }}^{n_{t}}[\left(x\left(i,t+l\right)-X_{i}\right)\times \left(s_{f}\left(u_{t}\right)-\bar{Y}\right)]}{{\left[\sum_{t=t^{\prime }}^{n_{t}}{\left(x\left(i,t+l\right)-X_{i}\right)}^{2}\times \sum_{t=t^{\prime }}^{n_{t}}{\left(s_{f}\left(u_{t}\right)-\bar{Y}\right)}^{2}\right]}^{0.5}}$$

where $X_{i}=\frac{\sum_{t=1}^{n_{t}}x\left(i,t\right)}{n_{t}};\bar{Y}=\frac{\sum_{t=1}^{n_{t}}s_{f}\left(u_{t}\right)}{n_{t}};t^{\prime }=\{\begin{array}{cc} 1 & l\geq 0\\ 1-l & l<0 \end{array};l=0,\pm 1,\pm 2\ldots$

The research index takes precedence when *l* < 0, the benchmark index takes precedence when *l* > 0, and the two indices synchronize when *l* = 0. *R*(*i*, *l*) is calculated for different *l*, then the *l* corresponding to the maximum |*R*(*i*, *l*)| is selected. If |*R*(*i*, *l*)| reaches a maximum when *l* = 0, the research index is synchronous. If |*R*(*i*, *l*)| reaches a maximum when *l* < 0, the research index is leading. Otherwise, it is lagging.

After classification, correlation coefficients need to be tested for significance. For a two-dimensional normal distribution, the probability density function of the correlation coefficient *r* is [30]:

(4) $$f_{n}\left(r\right)=\frac{n-2}{\pi }{\left(1-\rho^{2}\right)}^{\frac{n-1}{2}}{\left(1-r^{2}\right)}^{\frac{n-4}{2}}\int_{0}^{1}\frac{x^{n-2}}{{\left(1-\rho rx\right)}^{n-1}}\frac{dx}{\sqrt{1-x^{2}}}$$

According to sampling distribution theory [28], when:

(5) $$\left|r\right|>{\left|r\right|}_{\min}=\frac{t_{\alpha }}{\sqrt{t_{\alpha }^{2}+n-2}}$$

where we think the two are statistically significantly related; otherwise they are statistically independent of each other. In Equation (5), *α* is the level of significance; *t_α_* is the critical value for bilateral testing of the *t*-distribution with *n* − 2 degrees of freedom. 

### 3.2. Determining EWSI Warning Limits

Warning limits are hierarchical boundaries that represent severity. Firstly, using the following formula to perform threshold conversion on *s_f_*(*u_t_*) in Equation (1):

(6) $$y_{t}=0.5\times s_{f}\left(u_{t}\right)+0.5\;$$

Then, obtain the annual growth rate *r*(*s*, *t*) of EWSI*s* in year *t*.

(7) $$r\left(s,t\right)=\frac{x\left(s,\;\;t\right)-x\left(s,\;t-1\right)}{x\left(s,\;t-1\right)}\left(s=1,2,\ldots ,n_{s};t=1,2,\ldots ,n_{t}\right)$$

*r*(*s*, *t*) and *y_t_* are arranged from small to large, and they are fitted to the logical curve as follows:

(8) $$y_{t}=\frac{\theta }{1+e^{-k\left(r\left(s,t\right)-r_{c}\left(s,t\right)\right)}}$$

where *θ* is the upper limit of *y_t_*. Because *s_f_*(*u_t_*) ∈ [–1, 1] in Equation (1), *θ* = 1. *k* is the fitting coefficient. *r*_c_(*s*, *t*) is central value of *r*(*s*, *t*). It can be obtained when the second derivative of Equation (8) is equal to 0.

According to Equation (8), *r*(*s*, *t*) has been divided into three levels: for a positive index, when *r*(*s*, *t*) ≥ [*r*_c_(*s*, *t*) + *r*_2_(*s*, *t*)]/2, it is grade 1 (loadable); when [*r*_c_(*s*, *t*) + *r*_1_(*s*, *t*)]/2 ≤ *r*(*s*, *t*) < [*r*_c_(*s*, *t*) + *r*_2_(*s*, *t*)]/2, it is grade 2 (critical overload); when *r*(*s*, *t*) < [*r*_c_(*s*, *t*) + *r*_1_(*s*, *t*)]/2, it is grade 3 (overload). For negative indicators, the threshold is the opposite.

*r*_1_(*s*, *t*) and *r*_2_(*s*, *t*) above are obtained when the third derivative of Equation (8) is equal to 0:

(9) $$r_{1}\left(s,t\right)=r_{c}\left(s,t\right)-\frac{ln(2+\sqrt{3})}{k}$$

(10) $$r_{2}\left(s,t\right)=r_{c}\left(s,t\right)+\frac{ln(2+\sqrt{3})}{k}$$

### 3.3. AWI Design for EWS-RWRCC

To identify the EWSI and determine the EWS-RWRCC warning limits, we used the improved KLR model [31,32] to design the AWI, supplemented by different colored signal lights to judge the RWRCC trend.

Firstly, the EWSI is analyzed based on early warning performance as shown in Table 1. The number of times the corresponding event represented by ABCD in Table 1 occurred in *n_t_* is counted.

In Table 1, [B/(B + D)]/[A/(A + C)] represents the noise-to-signal ratio (NSR) [31]. Only when NSR < 1 can the index predict. In traditional method, the aggregate warning index is generally assigned by the NSR value as shown [31]:

(11) $${\mathrm{AWI}}_{t}=\frac{1}{n_{s}}\overset{n_{s}}{\underset{s=1}{\sum }}w_{s}\times V\left(s,t\right)=\frac{1}{n_{s}}\overset{n_{s}}{\underset{s=1}{\sum }}\frac{V\left(s,t\right)}{{\mathrm{NSR}}_{s}}$$

where *w_s_* is the weight of each EWSI*s*, AWI*_t_* is the aggregate warning index, and *V*(*s*, *t*) is the signal value of *s* at a given period *t*.

This paper used an analytic hierarchy process based on the accelerating genetic algorithm (AGA-AHP) [33] to determine *w_s_* in Equation (11) to improve the KLR model. According to the definition, smaller NSR values have relatively larger weights. Therefore, the reciprocal judgment matrix ***M*** can be obtained by:

(12) $$M={\left(a_{sj}\right)}_{n_{s}\times n_{s}}=\left[\begin{array}{ccc} a_{11} & \cdots & a_{1n_{s}}\\ \vdots & \ddots & \vdots \\ a_{n_{s}1} & \cdots & a_{n_{s}n_{s}} \end{array}\right]$$

where a_sj_ = NSR*_j_*/NSR*_s_*(*s*, *j* = 1, 2… *n_s_*).

Let the weight in ***M’*** be {*w_s_* | *s* = 1, 2… *n_s_*} too, then ***M’*** is ***M***’s optimal consistency judgment matrix when it can make the CIC(*n_s_*) from the following equation reach the minimum value [34]:

$$\min\mathrm{CIC}\left(n_{s}\right)=\frac{\sum_{s=1}^{n_{s}}\sum_{j=1}^{n_{s}}\left|b_{sj}-a_{sj}\right|}{n_{s}^{2}}+\frac{\sum_{s=1}^{n_{s}}\sum_{j=1}^{n_{s}}\left|b_{sj}w_{j}-w_{s}\right|}{n_{s}^{2}}$$

(13) $$s.t.\{\begin{array}{c} a_{ss}=1\\ \frac{1}{b_{js}}=b_{sj}\in \left[a_{sj}\left(1-d\right),a_{sj}\left(1+d\right)\right]\\ \overset{n_{s}}{\underset{s=1}{\sum }}w_{s}=1,w_{s}>0 \end{array}$$

where CIC(*n_s_*) is the consistency index coefficient; *d* is a non-negative parameter, which can be selected from [0, 0.5]. Obviously, smaller CIC(*n_s_*) values lead to greater consistency of ***M***.

The accelerated genetic algorithm (AGA) is a general global optimization method. It is relatively simple and effective for solving optimization problems. After a large number of numerical calculation experiments and referring to the relevant literature [10,34,35], the authors believe that when the value of CIC(*n_s_*) is less than 0.2, ***M*** can be considered to have satisfactory consistency, and the weights are acceptable.

After weights are determined by Equations (12) and (13), the following formula is used to build the AWI:

(14) $${\mathrm{AWI}}_{t}=\overset{n_{s}}{\underset{s=1}{\sum }}w_{s}\times V\left(s,t\right)$$

According to the principle of equal division, the *s_f_*(*u*) obtained by RWRCC evaluation was divided into five levels: inverse potential [−1.0, −0.6), partial inverse potential [−0.6, −0.2), symmetrical potential [−0.2, 0.2], partial identical potential (0.2, 0.6], and identical potential (0.6, 1.0] [10]. It can be seen that when *s_f_*(*u*) < −0.2, the results belong to a relative inverse condition. Since ${\mathrm{AWI}}_{t}\in \left[0,3\right],$ we chose the boundary value of warning to be three-fifths of 3 correspondingly. After dividing the remaining threshold equally, we can get 0 ≤ AWI*_t_* ≤ 1.8, no alarm (green); 1.8 ≤ AWI*_t_* ≤ 2.1, mild alarm (blue); 2.1 < AWI*_t_* ≤ 2.4, moderate alarm (yellow); 2.4 < AWI*_t_* ≤ 2.7, severe alarm (orange); AWI*_t_* > 2.7, very severe alarm (red).

### 3.4. Study Area

**Figure 2:**
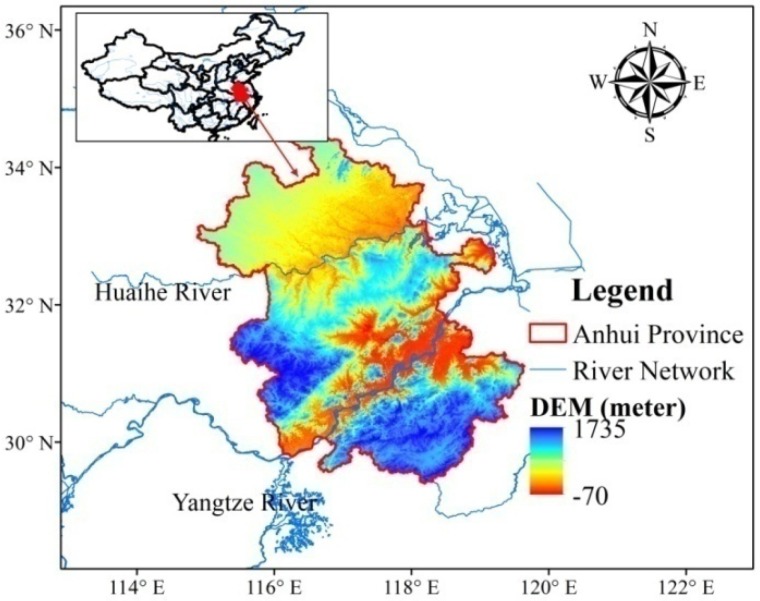
The location of Anhui province.

Anhui Province is in the southeast of China’s interior and at the middle and lower reaches of the Yangtze and Huaihe Rivers, at 29°41′–34°38′ N and 114°54′–119°37′ E (Figure 2). It extends across the Huaihe, Yangtze, and Xin’an Rivers and is 450 km wide from east to west and 570 km long from south to north, covering an area of 140,100 km^2^. The area has varied topography, including plains, hills, and mountains, which represent 24.1%, 28.9%, and 29.4% of the total, respectively. The landform in the area is composed of the Huaihe River basin, Jianghuai tableland and hills, the West Anhui hilly mountains, the Yangtze River plain, and the mountain area of South Anhui. The terrain gradually declines from the mountain core to the valley, and the mountains are mostly distributed in north–east and east–west directions.

Anhui Province is located in a midlatitude north–south climate transition zone. As it is affected by the monsoon, Anhui Province receives more precipitation in summer than in winter, and annual and interannual precipitation varies greatly. The multiyear average amount of water resources in the province for many years is 71.6 billion m^3^, ranking 14th in China. There is 1100 m^3^ water resource per capita, only about half the national average. Due to the uneven distribution of rainfall in time and space and the lack of water resources, it is becoming more and more difficult to meet the demand for economic development. Water resource shortages have become a constraint on the lives of urban residents in some parts of Anhui Province.

## 4. Example of Application

### 4.1. Identification of EWSI in Anhui Province

#### 4.1.1. Benchmark Index

**Figure 3:**
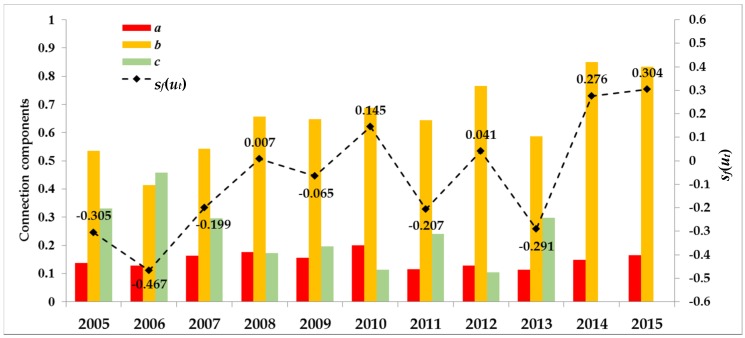
Connection numbers and corresponding *s_f_(u_t_)* values for evaluating regional water resources carrying capacity (RWRCC) from 2005 to 2015 in Anhui Province.

A diagnosis and evaluation model for RWRCC based on connection number was constructed by Li et al. [28], who assessed the overall RWRCC in Anhui Province. The results are shown in Figure 3.

As a whole, the results of the comprehensive evaluation of RWRCC in Anhui Province from 2005 to 2015 have obvious volatility, and the fluctuation range of *s_f_*(*u*) is between −0.5 and 0.4. Moreover, *s_f_*(*u*) values in 2005, 2006, 2011, and 2013 were close to −1, indicating that carrying capacity was worse in these years. However, *s_f_*(*u*) was closer to 1 in 2014 and 2015, indicating that the capacity tended to be improved.

#### 4.1.2. Early Warning Sign Index

RWRCC is a complex system composed of support (water resources system), pressure (economic, social, and ecological environment system), and regulation [36,37]. The EWSI can be chosen from indices for evaluation, but those with no significant change around the average value should be screened out. The early warning system needs to have certain change characteristics [38,39]. Twenty-five candidate indices for EWSI were established after consulting experts’ opinions and considering the sensitivity of each index to the RWRCC balance, as shown in Table 2.

In Table 2, water quantity refers to the water resources provided for maintaining social development, which is divided into average water supply to each person annually (X1) and water resources provided by surface water (X2). The water quality mainly reflects the water quality of national water functional areas and rivers related to drinking water (X3–X5). Economic development includes population and GDP, closely related to social economy (X6–X8) [40]. It also includes the proportion of water for agricultural production (X9). Water utilization and efficiency are mainly concentrated on daily life, industry, agriculture and ecology (X10–X17). Among them, water consumption of industrial added value (X12) is an indicator of energy management, which is generally reflected in the water resources bulletin. The matching degree of resources (X18–X19) reflects the corresponding situation of water resources, population, and farmland. The development degree of water resources (X20–X22) reflects the effective development and management of water resources in a certain region. The level of water supply (X23–X25) represents the capacity of the regional water supply system, which is related to the water safety in urban and rural areas.

The correlation coefficients *R*(*i*, *l*) when *l* = 0, ±1, and ±2 were obtained using Equation (3), according to the benchmark index in Figure 2 and candidate indices in Table 2. The maximum *|R*(*i, l*)*|* and corresponding *l* are shown in Table 3.

The significance of the correlation coefficients should be tested according to Equations (4) and (5) to determine the degree of relevance. In this study, the sample size *n* was 11. When 1 − *α* = 95%, *t_ɑ_* is 2.262, and by substituting it into Equation (5), we obtained an absolute value of 0.602 for the minimum correlation coefficient. Similarly, when 1 − *α* = 90%, *t_ɑ_* was 1.183, and the absolute value was 0.521. We believe that the absolute value should be greater than 0.6, considering the small sample size of this study. After screening absolute values of the correlation coefficients in Table 3, the early warning sign indices were obtained, as given in Table 4.

### 4.2. Determination of EWS Warning Limits in Anhui Province

**Figure 4:**
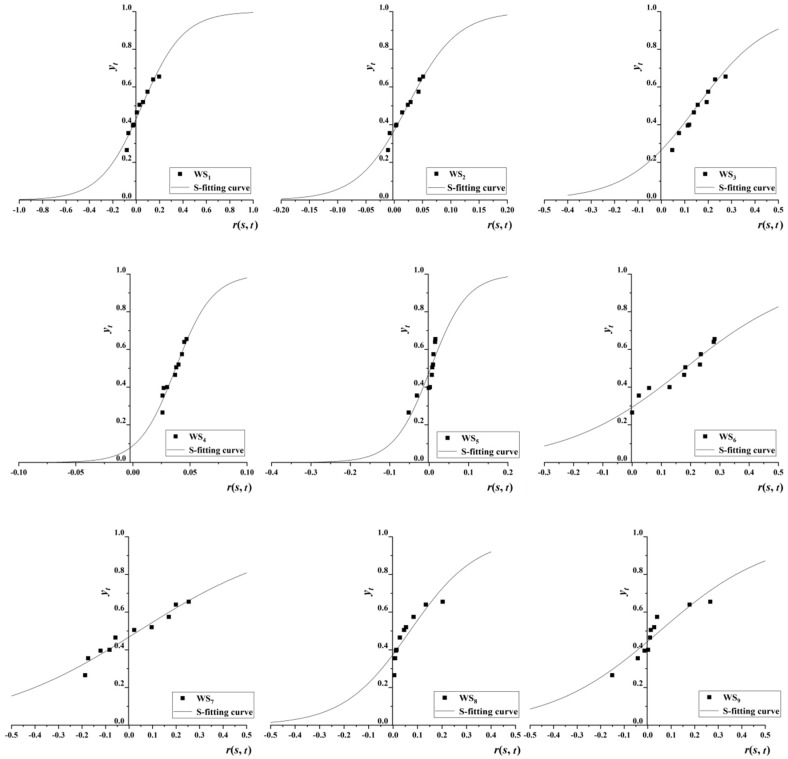
S-curves of *r*(*s*, *t*) and *y_t_* for EWS-RWRCC in Anhui Province.

Firstly, *s_f_*(*u_t_*) thresholds were changed to *y_t_* by Equation (6). Then, *y_t_* were arranged in increasing order, as were the EWSI annual growth rates from 2005 to 2015 shown in Table 4. Finally, *r*(*s*, *t*) and *y_t_* curves were fitted using the logical curve (also known as the S-curve), shown in Figure 4.

The *r*_c_(*s*, *t*), *r*_1_(*s*, *t*), and *r*_2_(*s*, *t*) S-shaped curve values were obtained by using Equations (9) and (10). Then, EWSI warning limits were classified according to index type and the corresponding grading method. The results are shown in Table 5.

As Figure 3 and Table 5 show, all indices had good fitted curves, with R^2^ values greater than 0.80, except for WS_5_, whose R^2^ value was 0.75. WS_3_ and WS_4_ in Table 4 belong to the pressure of RWRCC. Combined with Table 5, when the annual GDP per capita growth rate reached 25% or the annual population growth rate reached 5%, RWRCC in Anhui Province was close to the upper limit of critical overload. From the comparison of the two thresholds, it is clear that the RWRCC is more sensitive to the growth of population. WS_5_, WS_7_, and WS_9_ had negative critical overload upper limits. This showed that water resources had a buffer capacity for declines in these indices. For WS_7_ and WS_9_, both of which were within a certain range, the decrease will not have a strong impact. This means less sensitivity to these indexes. However, for WS_5_, the critical overload interval was relatively small. This shows that RWRCC is more sensitive to the decrease of WS_5_ when there is a certain decline space. This meant that, in the long run, to maintain the sustainable utilization of water resources it will still be necessary to ensure effective irrigation area growth value. The lower limits of WS_1_, WS_6_, and WS_8_ were positive. This reveals that RWRCC is very sensitive to the decline of these indicators. Under the current economic development situation, ensuring their continued growth can avoid negative impacts on RWRCC in Anhui Province.

### 4.3. AWI Design for EWS-RWRCC in Anhui Province

#### 4.3.1. EWSI Performance Analysis

EWSI values shown in Table 4 were analyzed according Table 1 in Section 3.3. According to the principle of equal sharing, when *s_f_*(*u_t_*) in Equation (1) was less than −0.2 it meant that a crisis occurs. When EWSI values were in critical overload and overload states, early warning signals were sent. The number of times an event represented by ABCD occurred in *n_t_* is shown in Table 6.

Ideally, a qualified early warning sign index would have both A and D values greater than 0 and B = C = 0 in Table 1. However, in reality, very few indices can completely satisfy this requirement, so other evaluation criteria needed to be defined. The conditional crisis probability A/(A + B) reflected the effectiveness of early warning as the proportion of correct signals. In Table 6, A/(A + B) values for all indices were between 30% and 60%, indicating that they were all effective at early warning. If NSR > 1, it meant that the index produced more erroneous signals than correct ones. In Table 6, only the NSR value for WS_2_ was greater than 1, and it had the lowest proportion of correct signals, so WS_2_ was excluded.

Based on the boundary conditions that NSR ≤ 1 and A/(A + B) > (A + C)/(A + B + C + D), WS_3_, WS_6_, and WS_8_ had better early warning capacity, which was consistent with their leading results from Table 4. Although WS_1_ was identified as a leading index in Table 4, its effectiveness in Table 6 was poorer than the other three, indicating that EWSI values identified by the data had different early warning capacities.

#### 4.3.2. The Aggregate Warning Index

To consider the signals provided by EWSI values for Anhui Province and to clarify the amount of pressure facing regional water resources, NSR values for each index in Table 6, except WS_2_, were substituted into Equations (12) and (13) to obtain weights *w_s_,* and warning signal values *V*(*s*, *t*) for 2006–2014 were obtained (Table 7). These were substituted into Equation (14) to obtain the aggregate warning index AWI*_t_* from 2006 to 2014. Further, AWI*_t_* values were colored according to the criteria in Section 3.3. A green light indicates a stable RWRCC, so a water conservancy department can continue development. Blue and yellow lights indicate slight changes in RWRCC, and a yellow light suggests that some importance should be attached to the critical status upper limit values. Orange and red lights warn that the RWRCC may be overloaded, indicating that relevant management departments must take measures to improve the status and cope with water utilization pressures.

Weight refers to the importance of an indicator relative to an object, emphasizing its contribution to the whole. From Table 7, the weights of WS_3_, WS_6_, and WS_8_ are larger, meaning that they are the key factors affecting AWI. The weight of WS_1_ is the smallest of all, which indicates that AWI is not comparatively sensitive to the change of WS_1_. Combining the sensitivity analysis of EWSI with RWRCC, the changes of WS_6_ and WS_8_ are the most sensitive factors affecting the early warning effect.

It can be seen in Table 7 that the Anhui Province signal was green in 2014, blue in 2009, 2011, and 2013, yellow in 2006–2008, and orange in 2010 and 2012. Yellow and orange lights accounted for more than half of the nine years between 2006 and 2014. Specifically, in Table 7, orange in 2010 and 2012 was consistent with the fact that *s_f_*(*u_t_*) values for 2011 and 2013 got close to −1 in Figure 2. Comparatively, the signal light was yellow in 2006, and in 2007, the status was better than the former two years. In 2010 and 2012 when *s_f_*(*u_t_*) values were greater than 0, the signal light was blue in 2009 and 2011. These all indicated that AWI was sensitive to tendency of RWRCC and had early warning capabilities to a certain extent. In 2013, the signal light was blue, and for 2007 and 2008 it was yellow. Taken together, AWI showed high alert when water resources status was in critical overload by sending a warning signal of worsening development. Therefore, it meets the strictest management system adopted for China’s water resources utilization.

## 5. Discussion

We believe that an appropriate benchmark index is the important basis for obtaining indices with effective early warning capacity when applying the TDCA method to EWS-RWRCC. The EWSI must be representative to reflect current RWRCC status. Moreover, the indices must be sufficiently sensitive to changes in carrying condition. Therefore, we chose the set pair potential based on subtraction in the SPA method. It successfully described the relative certain and uncertain systems.

RWRCC is an important part of resource and environmental carrying capacity. In recent years, with the rapid development of the social economy and the acceleration of industrialization, the impact of human beings on resources and the environment has also been increasing [41]. In view of the guiding significance of early warning of resource and environmental carrying capacity, relevant research has also attracted the attention of scholars and has carried out many beneficial explorations in the fields of theory [42,43], method [44,45], and regional demonstration. Generally speaking, the research focus has mainly been on the static analysis of the current capacity, which is not distinguished from the evaluation results. At the same time, the effective selection of early warning indicators, threshold standard division, and other issues need to be further studied. The study of economic early warning originated in the late 19th century and has become mature nowadays. For the early warning research of resource and environmental carrying capacity, it can be similar to the reference and improvement of the achievement of economic early warning (TDCA and KLR model) in this paper, so as to establish a more accurate and reasonable dynamic early warning model.

It is worth noting that determining early warning sign indices and limits is a dynamic process. According to the historical data, they can only maintain early warning effectiveness over a given time scale. This means that, with the passage of time and the changing environment, the carrying state of water resources develops constantly, and the relevant parameters for EWS-RWRCC need to be adjusted and revised.

## 6. Conclusions

EWSI can predict potential imbalances and emergencies, which is the basis of the EWS. In this paper, the candidate indices were classified by using the TDCA method and the set pair potential based on subtraction in the SPA method. The coincident and leading indices were used as EWSI values for RWRCC. A total of nine indices were obtained from early warning analysis of EWS-RWRCC in Anhui Province, including four leading indices. Of those, WS_1_ andWS_8_ represented the capacity of a region to supply domestic and production water, WS_3_ reflected the economic development status of a region, and WS_6_ reflected regional industrial production water use efficiency. Generally, these four indices covered the support, pressure, and regulation subsystems of RWRCC.

(1) The warning limits of EWSI are important criteria for judging the degree deviation, and index determination is a central focus of EWS. Classifying EWS-RWRCC warning limits based on S-curve was proposed in this paper. Relevant studies of Anhui Province showed that the annual growth rates of per capita GDP and the urban population percentages of 25% and 5% had an adverse effect on the RWRCC carrying status. The water resources carrying status in Anhui Province had some buffer capacity for drinking water source quality, proportion of effective irrigation, surface water control rates, and groundwater supply capacity. Increased water supply and repetitive use rate of industrial water should be guaranteed in the long run to maintain the sustainable utilization of water resources.

(2) AWI can be used to visualize the development trend. It is an important part of EWS. The AWI for EWS-RWRCC based on the improved KLR method was constructed. Index weights were determined using the AGA-AHP method to emphasize the theory’s scientific quality. The application results showed the AWI could describe interannual changes in RWRCC in Anhui Province. The index was applicable based on the strictest systems for managing water resources.

(3) In the early warning study of RWRCC, we found that the AWI had significant fluctuations during critical status from 2006 to 2014, and the RWRCC was poor. The average annual per capita GDP growth rate was about 16% over the past 10 years, and values for 2008, 2010, and 2011 were above 20%, exerting some adverse effects on the water resources carrying status in 2009, 2011, and 2013. Furthermore, water supplies in Anhui Province in 2010 and 2012 decreased from the previous years, which also affected carrying status in 2011 and 2013. Given the current situation, close attention should be paid to future population status and trends, GDP, and water supplies to monitor RWRCC in Anhui Province.

## Figures and Tables

**Table 1:** Performance criteria of early warning sign index (EWSI) for early warning system for regional water resources carrying capacity (EWS-RWRCC).

	Crisis Occurs in the Next Year	No Crisis Occurs in the Next Year
Index sends out signal	A	B
Index does not send out signal	C	D

**Table 2:** Candidate indices for EWS-RWRCC in Anhui Province.

Classification	Mark	Index	Calculation Method or Data Source
Water quantity	X_1_	Water supply per capita, m^3^	Water supply/population
	X_2_	Surface water supply, 100 million m^3^	Statistic yearbook
Water quality	X_3_	Compliance rate of national water function zone, %	Statistic yearbook
	X_4_	Compliance rate of drinking water source quality, %	Statistic yearbook
	X_5_	Rate of better river water quality (≥Grade III), %	Statistic yearbook
Economic development	X_6_	GDP per capita, 10,000 yuan	GDP/total population
	X_7_	Population density, person·km^-2^	Population/land area
	X_8_	Percent of urban population, %	Urban population/total population
	X_9_	Percent of agricultural water consumption, %	Agricultural water consumption/total water consumption
Water utilization	X_10_	Water consumption per capita, m^3^	Water consumption/total population
	X_11_	Water consumption per 10,000 yuan of GDP, m^3^	Industrial water consumption/GDP
	X_12_	Water consumption of industrial added value, m^3^	Statistic yearbook
	X_13_	Water consumption of farmland irrigation, m^3^	Statistic yearbook
	X_14_	Ecological water consumption, %	Statistic yearbook
Water efficiency	X_15_	Percent of effective irrigation, %	Effective irrigation area/cultivated area
	X_16_	Repetitive use rate of industrial water, %	Statistic yearbook
	X_17_	Water consumption rate, %	Statistic yearbook
Resource matching	X_18_	Water resources of farmland, m^3^/km^2^	Total water resources/cultivated area
	X_19_	Water resources per capita, m^3^	Total water resources/population
Development degree	X_20_	Annual water supply modulus, 10^4^ m^3^/km^2^	Annual water supply/land area
	X_21_	Control rate of surface water, %	Project water storage/surface water resources amount
	X_22_	Percent of water-saving irrigation, %	Water-saving irrigation area /effective irrigation area
Water supply level	X_23_	Average daily coefficient, m^3^/day	Statistic yearbook
	X_24_	Groundwater supply capacity, %	Groundwater supply/total water supply
	X_25_	Tap water use rate in rural areas, %	Statistic yearbook

**Table 3:** Identification and classification of early warning candidate indices in Anhui Province.

Index	|*R*(*i*, *l*)|	*l*	Index	|*R*(*i*, *l*)|	*l*
X_1_	0.412	0	X_14_	0.698	1
X_2_	0.603	−1	X_15_	0.713	0
X_3_	0.653	2	X_16_	0.669	−1
X_4_	0.775	0	X_17_	0.433	0
X_5_	0.602	1	X_18_	0.779	1
X_6_	0.664	−1	X_19_	0.736	1
X_7_	0.728	2	X_20_	0.635	2
X_8_	0.684	0	X_21_	0.602	0
X_9_	0.634	1	X_22_	0.701	1
X_10_	0.769	1	X_23_	0.651	−1
X_11_	0.371	0	X_24_	0.667	0
X_12_	0.462	0	X_25_	0.717	2
X_13_	0.736	2			

**Table 4:** Early warning sign indices for EWS-RWRCC in Anhui Province.

Classification	Mark	Type of Index
Water quantity	WS_1_ (X_2_)	Leading
Water quality	WS_2_ (X_4_)	Synchronous
Economic development	WS_3_ (X_6_) WS_4_ (X_8_)	Leading Synchronous
Water efficiency	WS_5_ (X_15_) WS_6_ (X_16_)	Synchronous Leading
Development degree	WS_7_ (X_21_)	Synchronous
Water supply level	WS_8_ (X_23_) WS_9_ (X_24_)	Leading Synchronous

**Table 5:** EWSI warning limits for EWS-RWRCC in Anhui Province.

Index	R^2^	*r*_c_(*i*, *t*)	*r*_1_(*i*, *t*)	*r*_2_(*i*, *t*)	Warning limits		
					Loadable	Critical	Overload
WS_1_	0.947	0.048	−0.186	0.283	≥0.09	(0.09, 0.01]	<0.01
WS_2_	0.963	0.004	−0.034	0.082	≥0.04	(0.04, −0.01]	<−0.01
WS_3_	0.920	0.154	−0.045	0.353	<0.05	[0.05, 0.25)	≥0.25
WS_4_	0.932	0.037	0.016	0.059	<0.03	[0.03, 0.05)	≥0.05
WS_5_	0.751	0.004	−0.057	0.065	≥0.03	(0.03, −0.06]	<−0.06
WS_6_	0.932	0.150	−0.089	0.450	≥0.25	(0.25, 0.05]	<0.05
WS_7_	0.941	0.041	−0.379	0.461	≥0.25	(0.25, −0.17]	<−0.17
WS_8_	0.800	0.102	−0.110	0.248	≥0.18	(0.18, 0.02]	<0.02
WS_9_	0.845	0.052	−0.255	0.360	≥0.21	(0.21, −0.10]	<−0.10

**Table 6:** Analysis of single EWSI values in Anhui Province.

Index	A	B	C	D	A/(A + B)	(A + C)/(A + B + C + D)	B/(B + D)/A/(A + C)
WS_1_	2	3	2	2	0.40	0.44	1.20
WS_2_	2	4	2	1	0.33	0.44	1.60
WS_3_	4	3	0	2	0.57	0.44	0.60
WS_4_	3	4	1	1	0.43	0.44	1.07
WS_5_	3	4	1	1	0.43	0.44	1.07
WS_6_	4	3	0	2	0.57	0.44	0.60
WS_7_	3	4	1	1	0.43	0.44	1.07
WS_8_	4	3	0	2	0.57	0.44	0.60
WS_9_	3	4	1	1	0.43	0.44	1.07

**Table 7:** Weights *w_s_*, warning signal values *V*(*s*, *t*), and AWI*_t_* values from 2006 to 2014 in Anhui Province.

Indexes	Weights	2006	2007	2008	2009	2010	2011	2012	2013	2014
WS_1_	0.0871	1	3	1	1	2	3	2	1	3
WS_3_	0.1739	3	3	3	2	3	1	2	3	1
WS_4_	0.0978	3	1	1	2	3	2	3	2	2
WS_5_	0.0978	3	1	2	3	2	3	1	2	2
WS_6_	0.1739	3	1	3	2	2	3	3	2	1
WS_7_	0.0978	1	3	3	2	2	1	3	2	2
WS_8_	0.1739	3	3	3	2	3	1	3	2	1
WS_9_	0.0978	1	3	2	1	3	3	3	3	2
AWI*_t_*		2.4	2.2	2.4	1.9	2.5	2.0	2.5	2.1	1.5
Signal light		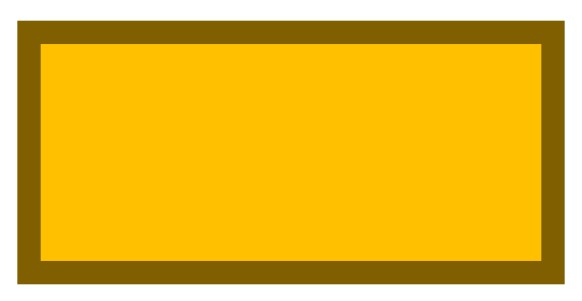	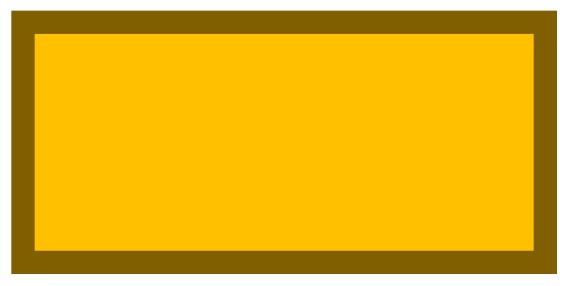	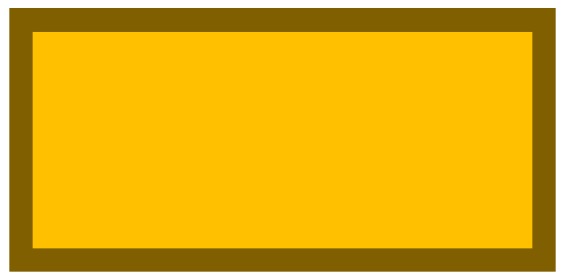	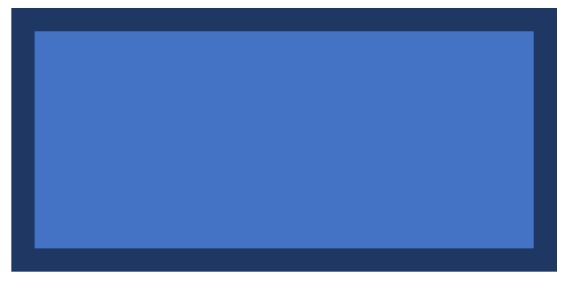	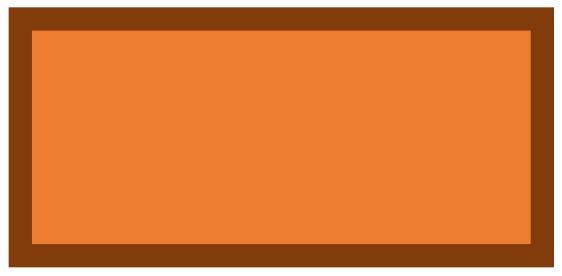	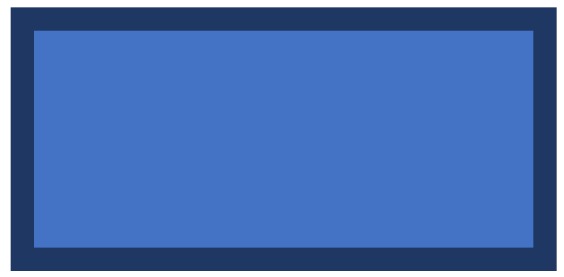	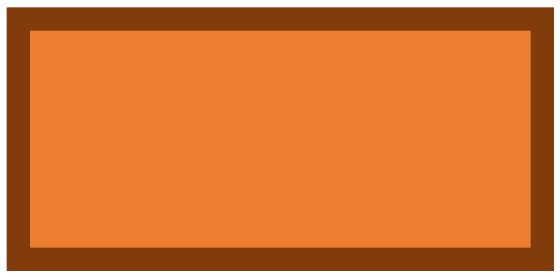	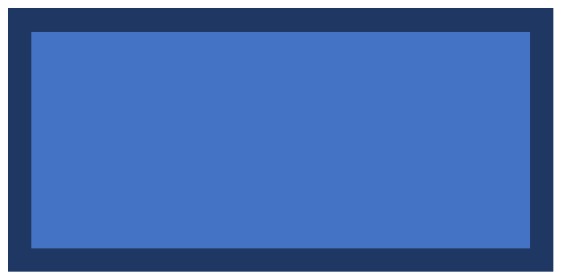	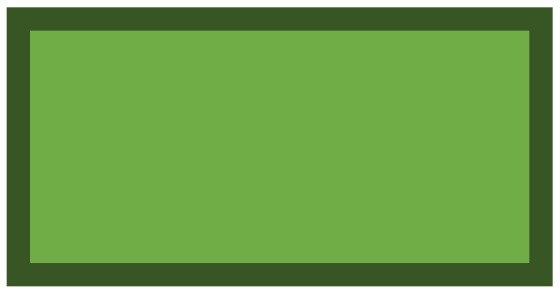

Here, 1, 2, and 3 represent loadable status, critical status, and overloaded status, respectively.
